# Evolutionary Changes after Translational Challenges Imposed by Horizontal Gene Transfer

**DOI:** 10.1093/gbe/evz031

**Published:** 2019-02-11

**Authors:** Stéphanie Bedhomme, Dolors Amorós-Moya, Luz M Valero, Nùria Bonifaci, Miquel-Àngel Pujana, Ignacio G Bravo

**Affiliations:** 1 CEFE, CNRS, Univ Montpellier, Univ Paul Valéry Montpellier 3, EPHE, IRD, France; 2 Experimental Molecular Evolution, Institute for Evolution and Biodiversity, Westfälische-Wilhelms Universität Münster, Germany; 3 Secció de Proteomica, SCSIE Universitat de Valencia, Spain; 4 ProCURE, Catalan Institute of Oncology (ICO), Bellvitge Institute of Biomedical Research (IDIBELL), Barcelona, Spain; 5 Centre National de la Recherche Scientifique (CNRS), Laboratory MIVEGEC (UMR CNRS, IRD, UM), Montpellier, France

**Keywords:** horizontal gene transfer, codon usage preferences, experimental evolution, antibiotic resistance, compensatory evolution

## Abstract

Genes acquired by horizontal gene transfer (HGT) may provide the recipient organism with potentially new functions, but proper expression level and integration of the transferred genes in the novel environment are not granted. Notably, transferred genes can differ from the receiving genome in codon usage preferences, leading to impaired translation and reduced functionality.

Here, we characterize the genomic and proteomic changes undergone during experimental evolution of *Escherichia coli* after HGT of three synonymous versions, presenting very different codon usage preference, of an antibiotic resistance gene. The experimental evolution was conducted with and without the corresponding antibiotic and the mutational patterns and proteomic profiles after 1,000 generations largely depend on the experimental growth conditions (e.g., mutations in antibiotic off-target genes), and on the synonymous gene version transferred (e.g., mutations in genes responsive to translational stress). The transfer of an exogenous gene extensively modifies the whole proteome, and these proteomic changes are different for the different version of the transferred gene. Additionally, we identified conspicuous changes in global regulators and in intermediate metabolism, confirmed the evolutionary ratchet generated by mutations in DNA repair genes and highlighted the plasticity of bacterial genomes accumulating large and occasionally transient duplications.

Our results support a central role of HGT in fuelling evolution as a powerful mechanism promoting rapid, often dramatic genotypic and phenotypic changes. The profound reshaping of the pre-existing geno/phenotype allows the recipient bacteria to explore new ways of functioning, far beyond the mere acquisition of a novel function.

## Introduction

Whereas in eukaryotes a large proportion of genetic novelty arises from gene duplications and mutations, an important part of the genetic diversity of prokaryotes is acquired through DNA transfer between species (Ochman et al. 2000; Koonin et al. 2001; Medini et al. 2005; Dagan et al. 2008). The large contribution of horizontal gene transfer (HGT) translates into dynamic bacterial genomes with frequent events of gene gains and losses. From an applied perspective, HGT is the main mechanism of propagation of enzymatic resistance to antibiotics, making it the biological mechanism at the origin of a fundamental public health problem (Martínez et al. 2015).

Even though it is a successful process from an evolutionary point of view, there are a number of biological and physical barriers to HGT (Thomas and Nielsen 2005). Following transfer, the maintenance of genetic information is not granted. It depends on the balance between the fitness advantage provided by the new gene(s) in the particular environment where the transfer took place and the cost linked to its expression and to the level of integration in the gene and protein networks of the receiving organism (Francino 2012; Baltrus 2013). When the balance leans toward maintenance of the transferred gene, compensatory evolution can take place to reduce the initial costs. HGT has long been perceived as a simple addition of new pieces and functions but recently two experimental evolution approaches (Lind et al. 2010; Bershtein et al. 2015) revealed compensatory evolutionary events triggered by the arrival in the genome of these new pieces. In these two studies, chromosomal genes were replaced by orthologs from other, mainly bacterial, species: orthologous ribosomal protein genes were transferred in *Salmonellatyphimurium* (Lind et al. 2010) and orthologous *folA* genes, encoding the essential metabolic enzyme dihydrofolate reductase were transferred in *Escherichiacoli* (Bershtein et al. 2015). The initial replacement incurred a cost, due mainly to the low quantity of the encoded protein. Experimental evolution lead to the increase in quantity of the protein expressed from the transferred gene, through duplications of the introduced gene (Lind et al. 2010), through mutations in the promoter of the introduced gene (Lind et al 2010; Bershtein et al. 2015) or through the insertion of an Insertion Sequence (IS) in the promoter of a gene maintaining proteostasis (Bershtein et al. 2015).

Three categories of HGT have been defined (Koonin et al. 2001): 1) acquisition of new genes, 2) acquisition of paralogs of existing genes, and 3) xenologous gene displacement whereby a gene is displaced by a horizontally transferred ortholog. The two experimental evolution approaches mentioned below analyzed evolution after transfers of the third category. Additionally, Lind et al. (2010) inserted the sequence of the orthologous genes without modification: both the amino acid sequence encoded and the codon usage of the gene vary across the genes inserted. Bershtein et al. (2015) made the choice to recode the orthologous genes using the highest frequency codon (defined in *E. coli folA* gene) for each amino acid, such that the different orthologous genes differ only in their amino acid sequence and not in their codon usage. The present study is focusing on post-HGT evolution in a context that differs from previous studies by at least two aspects: the category of HGT—the acquisition of a new antibiotic resistance gene was mimicked—and the maintenance of the same amino acid sequence while varying the codon usage preferences (CUPs). The match in CUP between the transferred gene and the receiving genome is indeed one of the key determinants of gene expression. CUPs are defined as deviations from the equal use of all codons within a synonymous family and is known to vary between genomes (Sharp and Li 1987; Hershberg and Petrov 2008). In organisms reproducing fast and in large populations, preferred codons usually correspond to transfer RNAs (tRNAs) with the highest gene copy number in the genome (Duret 2000; Lithwick 2003; Rocha 2004; Higgs and Ran 2008) and consequently to the tRNA with the highest concentration within a cell (Dong et al. 1996). CUP and tRNA copy number are proposed to have coevolved to optimize translation efficiency, translation accuracy, and protein folding (Sharp et al. 2010; Gingold and Pilpel 2014). High concentration of specific tRNAs ameliorates ribosomal processivity by increasing the acceptance rate of the cognate aa-tRNA, thence improving translation efficiency. Conversely, the use of rare codons slows down translation (Sørensen et al. 1989; Buchan 2006; Hershberg and Petrov 2008; Qian et al. 2012) and increases the probability of introducing erroneous amino acids (Schimmel 1989; Calderone et al. 1996; Kramer and Farabaugh 2006; Aguirre et al. 2011). Finally, a mismatch in CUP leads to higher amounts of misfolded proteins for two reasons. First, the insertion of erroneous amino acids can affect the secondary structure of the protein. Second, by changing translation speed, a mismatch in CUP can alter the kinetic coupling of translation speed and protein folding (Thanaraj and Argos 1996; Zhang et al. 2009; O’Brien et al. 2012; Spencer et al. 2012). A mismatch in CUP will thus have overall negative fitness effects through synthesis of low quantity of functional protein, consumption of energy and resources for the production of useless proteins, accumulation of misfolded protein, and clean-up costs (Drummond and Wilke 2008). The link between CUP mismatch and fitness might actually be more complex than assumed in the translational selection hypothesis. For example, fine modeling of translation suggest a trade-off between nonsense and missense error rate (Shah and Gilchrist 2010). Moreover, changes in codon usage of specific sequences not only modify their own translation but may also have significant impact on a large fraction of the proteome, through competition for the tRNA pool (Frumkin et al. 2018).

When a gene acquired through HGT and presenting a mismatch in CUP is maintained in the receiving genome, two nonexclusive evolutionary outcomes have been proposed. The first one is an amelioration process, that is, the accumulation of synonymous mutations driving the CUP of the transferred gene closer to the CUP of the receiving genome (Lawrence and Ochman 1997, 1998; Garcia-Vallve 2003). The second one is the selection for compensatory mutations outside the transferred gene, ultimately streamlining its expression (Mozhayskiy and Tagkopoulos 2012). At least four types of compensatory mutations, potentially increasing the production of functional protein, can be predicted: 1) mutations in the regulatory sequence of the introduced gene, 2) changes in the regulatory network that controls gene expression, 3) changes in the translation machinery (e.g., changes in tRNA gene copy number, or ribosomal mutations), and 4) changes in the posttranslational machinery (e.g., increase in chaperone production). The first two increase the transcription and/or translation level and the last two increase translation accuracy and efficiency and reduce the amount of misfolded protein.

In previous studies, we mimicked the acquisition by horizontal transfer of genes presenting different levels of CUP mismatch by introducing on a plasmid three synonymous versions of the chloramphenicol acetyl transferase (*cat*) gene in *E.coli*. CUP mismatch had a clear cost in terms of chloramphenicol resistance and thus of fitness in presence of chloramphenicol: the version with the best match in CUP provided the highest resistance whereas the two versions with CUP mismatch had a 3.5-fold and 20-fold reduction in Inhibitory Concentration 50 (IC50) calculated on growth rate (Amorós-Moya et al. 2010). These differences disappeared rapidly when populations carrying these gene versions were evolved experimentally. All populations evolved in chloramphenicol recovered a high resistance level after 400 generations, whereas populations evolved in ampicillin lost chloramphenicol resistance after 1,000 generations (Amorós-Moya et al. 2010; Bedhomme et al. 2017). Additionally, both the chromosome and the plasmid contributed to this compensatory evolution (see fig. 6 in Amorós-Moya et al. 2010 and fig. 3 in Bedhomme et al. 2017), with a higher contribution of the evolution of the plasmid when the initial cost of CUP mismatch was higher. The coding sequence of the introduced gene presented very few mutations under any condition and no amelioration process was observed. Instead, all populations evolved in chloramphenicol acquired small indels upstream the start codon of the gene that affected the translation level of the resistance protein and populations evolved in ampicillin presented diverse genetic changes leading to strong reduction of the *cat* gene expression (Amorós-Moya et al. 2010; Bedhomme et al. 2017). In the present study, genomic and proteomic approaches on the experimentally evolved populations are used to answer two questions: what is the genetic basis of post-HGT compensatory evolution?; how is the cell functioning affected by this compensatory evolution?

## Materials and Methods

The design and characteristics of the three synonymous versions of the *cat* gene and the derivation of the experimentally evolved populations have been described in details elsewhere (Amorós-Moya et al. 2010) and are summarized in supplementary figure S1, Supplementary Material online. Briefly, one *cat* version uses preferred codons for each amino acid based on the average codon usage of a set of highly expressed genes in *E. coli* (Opt-*cat*), another one uses nonpreferred GC-rich codons (GC-*cat*), and the last one uses nonpreferred AT-rich codons (AT-*cat*). These three gene versions were cloned under the control of a constitutive promoter in a classical cloning vector (pUC57), which additionally carries a *bla* gene, conferring resistance to ampicillin. The plasmids were transformed in *E. coli* top10 (Invitrogen). From each of the three populations carrying one of the synonymous versions of *cat*, six subpopulations were derived each one from a single colony and experimentally evolved for 1,000 generations: three of them in presence of 25 μg mL^−1^ of chloramphenicol and three in presence of 20 μg mL^−1^ of ampicillin.

### Whole Genome Sequence of Evolved Populations

Total DNA was extracted from the ancestral population and from the 18 evolved populations at generation 458 and 1,000 from 2-mL overnight cultures with the REALPURE extraction kit following manufacturer instructions. DNA samples were sequenced by Illumina Miseq at FISABIO (Valencia, Spain). The fastq files were cleaned using prinseq (Schmieder and Edwards 2011): the first 11 bp of each read were removed, reads with undetermined bases and with low quality scores were eliminated. Mapping and mutation identification was done using the breseq pipeline (Deatherage and Barrick 2014), with the *polymorphism detection* option, as the sequencing was done at the population level. Reads were mapped on the published genome of *E.coli* strain K12 substrain DH10B (GenBank accession number: CP000948.1). The only difference between the ancestral genome and the reference sequence (one additional IS10 insertion) was subtracted from the mutations identified in the evolved populations. The breseq output was cleaned manually to remove false polymorphisms due to mapping errors between high homology zones. By sequencing duplicate samples of the same populations, it was established that chromosomal mutations identified with a frequency above 0.2 were reliable. All mutations detected with a frequency above this value were kept in the data set.

### Identification of Mutational Patterns

Mutations in the genome may be adaptive or neutral, but maintained in the population because of drift or of background selection. To test whether the pattern of mutations was partially determined by the *cat* gene version carried and the antibiotic evolution treatment, permutational analysis of variance (Zapala and Schork 2006) was used. For each gene, every population was scored as being mutated (1) or not mutated (0). A permutation analysis was then conducted using *adonis* (vegan package in R v2.4-6), based on pairwise Jaccard distances to assess whether the mutation patterns were more similar within than between groups, groups being defined by the *cat* gene version, the antibiotic evolution treatment and their interaction. We then used parallel evolution as a criterion for adaptive evolution: the probability that a mutation spread by chance (i.e., without providing any fitness advantage) in two independent populations is very low (Lang and Desai 2014). The premise of parallel evolution was applied at the gene level. To do so, the list of mutated genes of the 18 evolved populations at generation 1,000 were analyzed with the Venndiagram package in R, to find genes or groups of genes sharing the same patterns of presence/absence of mutations among populations. A nominal logistic regression was performed on each of the lines of this table with gene (AT-, GC-, or OPT-*cat*), selection (Amp, Cam) and their interaction as factors and the presence/absence of a mutation in the gene as a response.

### Proteomic Analyses

The impact at the proteome level of the introduction of synonymous *cat* genes with different CUPs and of 1,000 generations of experimental evolution was analyzed using Isobaric Tag for Relative and Absolute Quantitation (Ross et al. 2004). This technique allows for the relative quantification of expressed proteins between one reference sample and up to seven test samples. Differential quantitative proteomics experiments were organized in two arms, each of them comparing the proteomes of eight populations. The first analyses were designed to compare the proteomic profiles of the initial populations with those of the experimentally evolved ones: the samples analyzed were from the initial populations carrying the three *cat* versions and from pools of the three evolved (g1000) populations for each *gene version* × *selection* treatment except populations carrying the AT-*cat* and evolved in chloramphenicol, because of methodological constraints. The AT-*cat* g0 served as reference for comparison purposes. The second analyses were designed to compare the proteomic profiles of populations carrying the AT-*cat* evolved under chloramphenicol with those of ancestral bacteria transformed with the corresponding evolved plasmids; the initial population (AT-*cat* g0) was compared with 1) a pool of the three populations evolved for 1,000 generations in ampicillin (AT-*cat* Amp), 2) the three populations evolved in chloramphenicol (AT-*cat* Cam1, AT-*cat* Cam2, and AT-*cat* Cam3), and 3) three populations of the ancestral bacteria (Top10) transformed with the plasmid extracted from the three previous populations (AT-*cat* Cam1^tr^, AT-*cat* Cam2^tr^, and AT-*cat* Cam3^tr^). We chose again the initial AT-*cat* g0 population as a reference for comparison. Each population was cultured in the conditions of selection for evolved populations and in 20 μg mL^−1^ ampicillin for initial populations. Proteins were extracted, trypsin digested, and the N-termini were covalently labeled with one of the eight available mass-reporters. Samples of a same experiment were then pooled, fractionated by nanoliquid chromatography, and analyzed by tandem mass spectrometry (MS/MS). Fragmentation data were then submitted to a database search to identify the labeled peptides and hence the corresponding proteins. A detailed procedure is given in the supplementary material, Supplementary Material online. For the analysis of the differential quantitative proteomics data, AT-*cat* g0 was used as the reference for ratio calculation in both experiments. First, to gain an overview of the shift trends on the different proteomes, we performed a cluster analysis of protein fold changes. Peptide data with variation above 1.25-fold relative to the control in at least one comparison were used in unsupervised hierarchical clustering analyses. Data were not standardized and the complete linkage method and Euclidean metric distance was applied. Second, a functional enrichment analysis was performed using Gene Ontology (GO) Biological Processes term and the Kyoto Encyclopedia of Genes and Genomes (KEGG) pathway annotations. Significance was assessed by comparing the observed frequency of each term/pathway in the test cluster with the expected frequency considering only those peptides that were measured in each differential proteomics assay. The *P* values were adjusted for multiple testing by applying a False Discovery Rate (FDR) threshold <5%. To assess potential bias of GO or KEGG annotation distributions in differential proteomics results (considering all peptide ratios relative to the reference), the Gene Set Enrichment Analysis tool (Subramanian et al. 2005) was used with default values for all parameters. Third, we focused on the raw results for the two antibiotic resistance proteins CAT and BLA, comparing protein levels between *cat* gene versions immediately after transfection, as well as after antibiotic selection (described in depth in Bedhomme et al. 2017). Finally, we analyzed the cellular levels of selected protein functional groups involved in protein synthesis and protein quality control, for which changes in the expression level could be expected to evolve in the presence of a codon usage biased gene (proteins of the chaperone system, ribosomal proteins, translation factors, and aminoacyl-tRNA synthetases).

## Results

### Experimental Evolution after Artificial HGT Leads to a Diversity of Genetic Changes

**Fig. 1.**
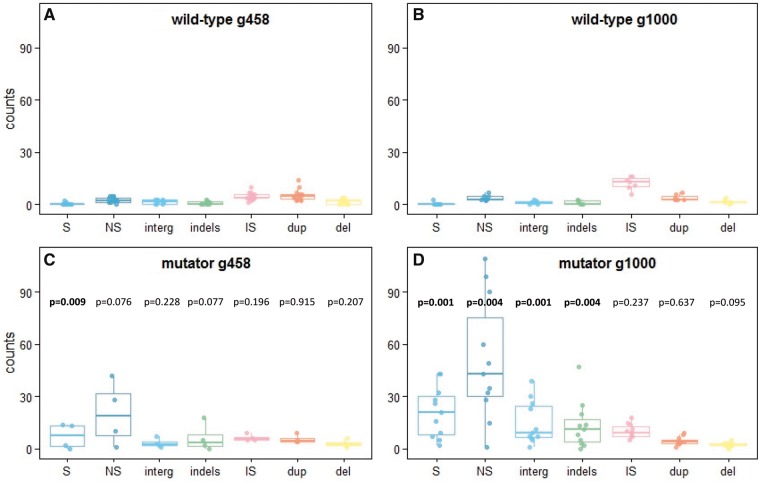
—Mutation spectra in evolved populations. Number of events for different mutation types per population, at generations 458 (*A*, *C*) and 1,000 (*B*, *D*) for wild-type (*A*, *B*) and mutator (*C*, *D*) populations. In blue, SNP (“S,” synonymous; “NS,” nonsynonymous; and “interg,” intergenic); in green, small insertions and deletions (<30 bp); in pink, insertion sequences; in orange, duplications; and in yellow, deletions. The bar depict the median, boxplots show first and third quartiles, and whiskers span the 95%. *P* values correspond to Mann–Whitney tests comparing the number of same type mutations in wild-type and mutator populations for a same generation.

The genomes of the 18 evolved lines were sequenced at population level at generations 458 and 1,000. Median chromosomal coverage was 63 (min: 33; max: 120). Coverage data were analyzed by a sliding window method to identify potential large duplications and deletions. The complete list of mutations detected is provided in supplementary table S1, Supplementary Material online. The number of mutations per population presented a bimodal distribution. Populations with a high number of mutations had always acquired disruptive mutations in one of the methyl-mismatch repair system (MMRS) genes *mutL* or *mutS* (supplementary table S1, Supplementary Material online). In all cases, these disruptive mutations corresponded to IS10 insertion events. Disruptive mutations in MMRS genes are known to trigger an increased mutation rate (Sniegowski et al. 1997) and to result thus in a mutator phenotype. In total, among the 18 experimentally evolved lines, 4 had become mutators by g458 and 11 by g1000 (supplementary table S1, Supplementary Material online). Disruption of *mut* genes was associated with an increased number of substitutions and small indels (particularly 1-bp indels; fig. 1). At g1000, mutator populations had accumulated over 15 times more mutations in coding regions than wild-type (67 [29.6–148] vs. 4 [2.3–9.2]; median [0.05–0.95 quantiles]; *P* = 10^−3^, Wilcoxon–Mann–Whitney test), and the fraction of synonymous mutations in mutators was higher than in wild-type (0.30 [0.21–0.51] vs. 0 [0–0.21]; median [0.05–0.95 quantiles]; *P* = 2.3 × 10^−3^, Wilcoxon–Mann–Whitney test; null expectation for *E. coli* 0.190). The susceptibility of the mismatch repair genes to undergo IS-mediated disruption not only paves the way to an increase in mutation and in substitution rates but also allows subsequently mutated genes to explore a different sequence space: although transitions represent 59% of mutations in coding sequences in nonmutators (42–74, 95% CI), they increase to 94% (90–96, 95% CI) in *mutS*-disrupted populations and to 97% (94–98, 95% CI) in *mutL*-disrupted populations. Similarly, 64% (46–79, 95% CI) of mutations in nonmutator populations lead to an increase in GC content, compared with 85% (81–89, 95% CI) in *mutS*-disrupted populations and to 76% (71–80, 95% CI) in *mutL*-disrupted populations. These numbers are in agreement with recent reports on the impact of mutators on the mutational spectrum (e.g., Couce et al. 2017).

**Table 1:** List of Genes Mutated in Five or More Populations at Generation 1000

Mutated gene		Antibiotic	Gene Version			Amino Acid Change or Mutation; Protein Length (p.l.)	Protein Function
			AT	GC	OPT		
**Significant effect of *antibiotic***	*adhE*	AMP				A392V(3), L571V, F583L(3), A626V, A626T, D672N; p.l.: 891 aa	Fused acetaldehyde-CoA dehydrogenase and iron-dependent alcohol dehydrogenase—inhibited by chloramphenicol
		CAM					
	*arcA*	AMP				I55V, R67C, N85D, N85H, V87A, E94G, E86G, D99G(3), N106T, T126A; p.l.: 239 aa	Global regulator. Regulates genes involved in redox metabolism
		CAM					
	*acrB*	AMP				IS10 2, ins 1 bp 73(2), A286D, IS1 3049; p.l.: 1,050 aa	Component of a multidrug efflux pump
		CAM					
	*sucC*	AMP				W248R, V263V, G292A, E350G(3), Q247R, A354V, L255R, I259S; p.l.: 388 aa	Succinyl CoA synthase beta-subunit
		CAM					
	*fabR*	AMP				T46A, insT 534, IS2 42, IS10 124(2), IS2 366, IS150 557; p.l.: 216 aa	Fatty acid biosynthesis regulator
		CAM					
**Significant effect of *gene version***	*spoT*	AMP				S103P, H179R, Y286H, G314S, Y391C, N456H; p.l. 702 aa	Guanosine-3′,5′-bis(diphosphate) 3′-pyrophosphohydrolase (both synthesizes and hydrolyzes the alarmone [p]ppGpp)
		CAM					
	*fadE*	AMP				Y158H, E323K(2), Y713H; p.l.: 814 aa	Fatty acid degradation, starvation inducible
		CAM					
	*fruA*	AMP				S86P, A431V, C472R, Y548D, IS10 1387, del 1bp 829, ins 1 bp 1382, IS10 1387, del 1bp 1492; p.l.: 564 aa	Fructose permease (membrane spanning protein)
		CAM					
	*mdoB*	AMP				IS10 1870(4), T > C 317; pseudogene length: 2,009 nt	Pseudogene
		CAM					
	*putP slt*	AMP				putP (D55G[2], I254V, I435F; p.l.: 502 aa), slt (G198D, ins 1 bp 275, ins 1 bp 1223; p.l.: 646 aa)	PutP: Na+/l-proline transporter; Slt: soluble lytic transglycosylase, cleaves murein residues
		CAM					
^a^	*rpsG*	AMP				Y154C, L157*(2); p.l.: 179 aa	30S ribosomal subunit protein S7
		CAM					
	*mutL*	AMP				IS10 977(3), IS10 1423(2); p.l.: 616 aa	Methyl-directed mismatch repair protein
		CAM					
	*cytR*	AMP				Y53D, del 1 bp 138, IS4 142(2), L204P, A257P, IS10 292, IS10 624 (2); p.l.: 342 aa	Antiactivator (TF) for nucleoside utilization regulon. Controls genes involved in (deoxy)nucleoside uptake and metabolism
		CAM					
	*mutS*	AMP				IS10 1552(2), IS10 1999(2), IS10 2463(2); p.l.: 854 aa	Methyl-directed mismatch repair protein
		CAM					
	*clpX ydeK*	AMP				*clpX* (T199A[2], A210V, I301V, Y385C; p.l.: 424 aa), *ydeK *(insC 781, del 1 bp 3298, del 1 bp 3962, insC 3839; p.l.: 1326 aa)	ClpX: ATPase subunit of ClpXP protease, molecular chaperone; *ydeK*: reconstructed pseudogene
		CAM					
	*yejG*	AMP				S25P, D89G(2), L61P, ins 8 bp 64(2); p.l.: 115 aa	Unknown function
		CAM					

Note.—The first column indicates the result of the nominal logistic regression (see Material and Methods). Shades of blue in the third column reflect whether 0, 1, 2, or 3 of the replicate populations in each *gene version* × *antibiotic* combination were mutated. The fourth column gives the position of the mutations within the protein, with either the corresponding amino acid change, the small indel, or the IS insertion event. Numbers in parentheses/brackets indicate the number of bacterial populations in which the indicated mutation was found. The fifth column describes the function of the protein. “*p.l.*,” protein length.

^a^ Significant effect of the interaction “*gene version* × *antibiotic.*”

Experimental evolution of a clonally reproducing organism followed by whole genome sequencing renders a list of mutations, some of which are possibly adaptive and some others are neutral or even slightly deleterious and may have increased in frequency as passengers of the adaptive ones. This process of genetic draft (Desai et al. 2007) is reinforced in mutator populations, with a higher proportion of passengers and a lower proportion of drivers (Couce et al. 2017). However, permutation ANOVA (ANalysis Of VAriance) revealed that populations either carrying the same *cat* gene version, or evolved in presence of the same antibiotic treatment, displayed a more similar pattern of mutated genes (model *R*^2^ = 0.34; *gene version*: pseudo-*F*_2,__12_ = 1.356, *P* = 0.002, *R*^2^ = 0.148; *antibiotic*: pseudo-*F*_1,__12_ = 1.412, *P* = 0.002, *R*^2^ = 0.077) although these factors explained only a modest fraction of the variation in these patterns. We then used parallel evolution at the gene level to identify mutations with a higher probability of being adaptive, with the rationale that if a gene has been mutated and the mutation retained in more than one population evolving in the same conditions, the likelihood of this parallelism of being due to selection is high (Dettman et al. 2012). Heterogeneity in mutation rates and in selection intensity both contribute to parallel evolution, but in bacteria heterogeneity in selection intensity has been shown to play a highly preponderant role (Bailey et al. 2017). Table 1 and supplementary table S2, Supplementary Material online, list all genes mutated in at least two populations.

### Ambiguous Adaptive Value of Large Genomic Duplications

**Fig. 2.**
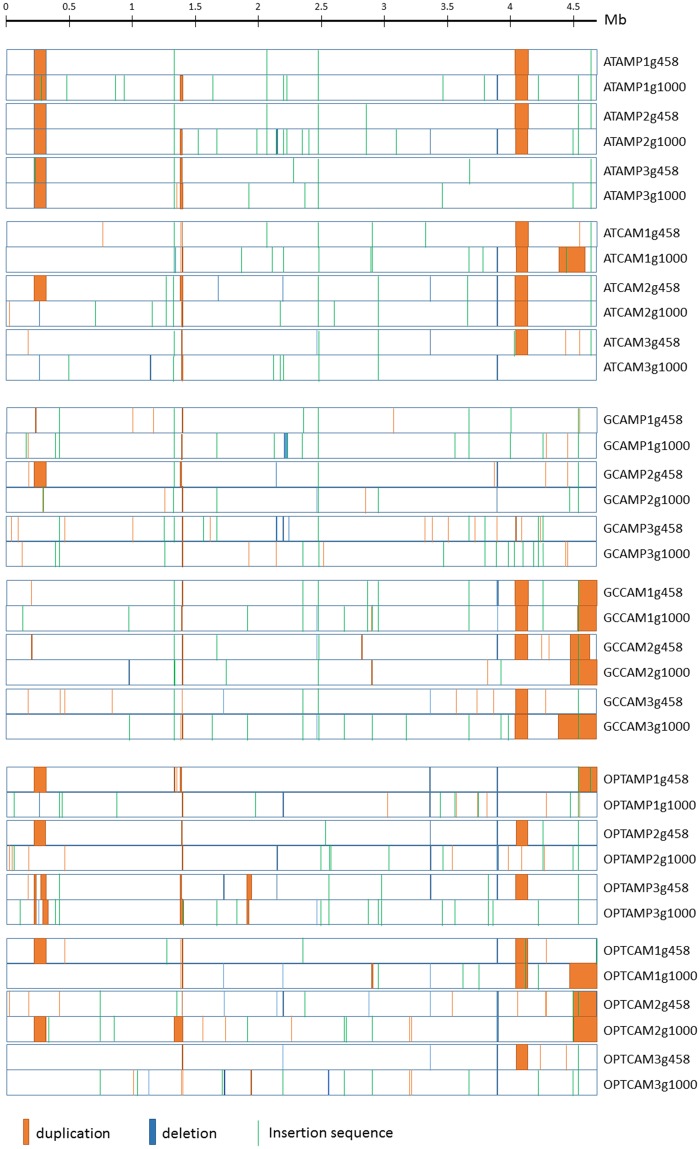
—Position and size of the duplications, deletions, and new insertion sequences in the 36 evolved genomes sequences.

Coverage data analysis revealed that large chromosome stretches ranging in size from 1 to 300 kb were often duplicated (fig. 2). Some deletions were also identified, albeit of smaller length, and detected in a lower number of populations (fig. 2). Permutation tests on the patterns of deletions and duplications revealed a significant effect of the *cat* gene version and antibiotic of selection (model *R*^2^ = 0.43; *gene version:* pseudo-*F*_2,__12_ = 1.874, *P* = 0.004, *R*^2^ = 0.179; *antibiotic*: pseudo-*F*_1,__12_ = 2.426, *P* = 0.001, *R*^2^ = 0.116). When the pattern of duplications was analyzed separately, both effects were maintained (model *R*^2^ = 0.44; *gene version*: pseudo-*F*_2,__12_ = 1.671, *P* = 0.034, *R*_2_ = 0.155; *antibiotic*: pseudo-*F*_1,__12_ = 3.174, *P* = 0.001, *R*^2^ = 0.147) whereas when the pattern of deletions was analyzed separately, only the *cat* gene version had a borderline significant effect (model *R*^2^ = 0.33; *gene version*: pseudo-*F*_2,__12_ = 1.646, *P* = 0.048, *R*_2_ = 0.183; *antibiotic*: pseudo-*F*_1,__12_ = 0.929, *P* = 0.48, *R*^2^ = 0.052).

**Fig. 3.**
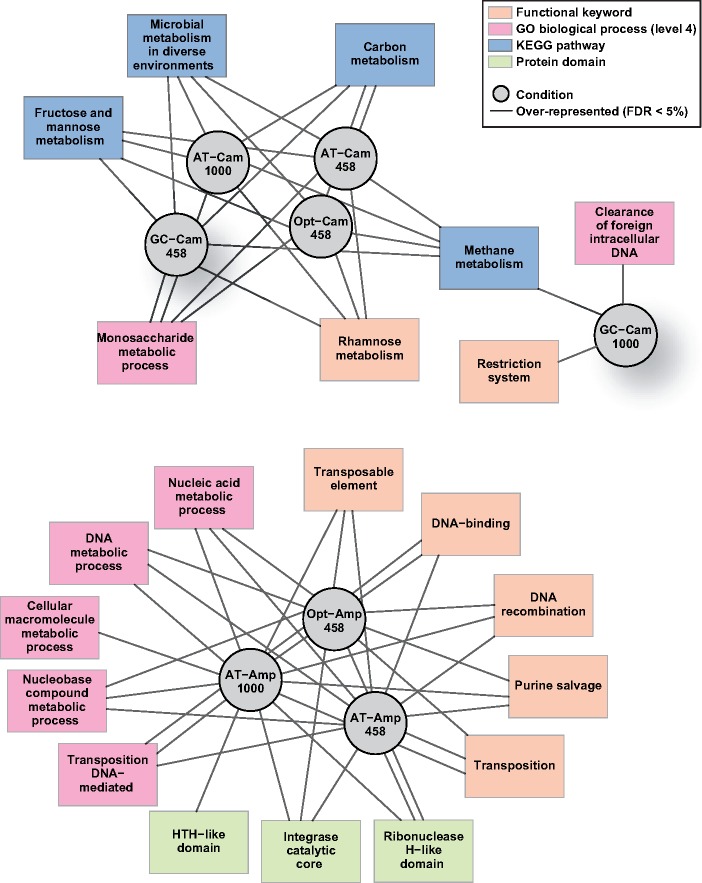
—Functional enrichment analysis for genes present in duplicated genomic stretches. Gene functions were searched from annotations in the KEGG and in the GO repositories. Gray lines display the overrepresentation of genes in a given functional category in the corresponding duplicated genomic stretches of a bacterial population. A line linking a category to a given population reflects enrichment of this functional category in this population. The sharp split between populations selected in chloramphenicol and in ampicillin is a result of the analysis and has not been enforced.

The role of large genomic alterations was evaluated by a functional enrichment analysis relative to the reference genome. The list of genes in deleted regions (in at least two sister populations) did not provide sufficient power to identify functional enrichments. Duplicated regions tended to be enriched in a number of functional categories independently of the selection conditions and of the *cat* version. This was the case of two-component regulatory systems or of carbohydrate catabolic processes. Enrichment analyses indicated some population differentiation based on the antibiotic selective pressure, with a sharp cleavage between populations selected under chloramphenicol and under ampicillin (fig. 3). Duplicated regions in populations evolved under ampicillin appeared to be specifically enriched in genes involved in amino acid and nitrogen base–related metabolism. The connection between the enrichment in these functional categories and the specific selection pressures in our experiment is not obvious and may be a mere correlate of chromosomal location of genes in genomic stretches with increased propensity to duplication, as suggested by parallelism in duplicated regions (fig. 2).

In addition to examining functional categories, we analyzed whether large duplications and deletions had an effect on the genomic tRNA gene content. This was indeed the case in 32 out of the 36 evolved genomes. We next tested whether these alterations improved the match between the tRNA pool and the CUP of transferred *cat* version. Because the use of rare codons is known to be a key determinant on translational speed and fidelity, we analyzed the variation in copy number of tRNAs able to translate rare codons (rare defined by their frequency in the *E. coli* genome). By design, the set of rare codons used in AT-*cat* and in GC-*cat* was virtually nonoverlapping, and Opt-*cat* did not use any rare codon. None of the tRNAs decoding a rare codon used in AT-*cat* was affected by a copy number change during the experimental evolution of the populations carrying this version of the gene. In three sequenced genomes of evolved populations carrying the GC-*cat*, the only tRNA gene that underwent duplication was one decoding a rare codon (ACG, Thr). In all other genomes of evolved populations carrying GC-*cat*, the proportion of changes in tRNA gene copy number affecting a tRNA decoding a rare codon was lower or equal to the one expected by chance (assuming an equal probability of duplication for each tRNA gene, see supplementary table S3, Supplementary Material online).

### Experimental Evolution after HGT Induces Global and Specific Changes in the Proteome

**Fig. 4.**
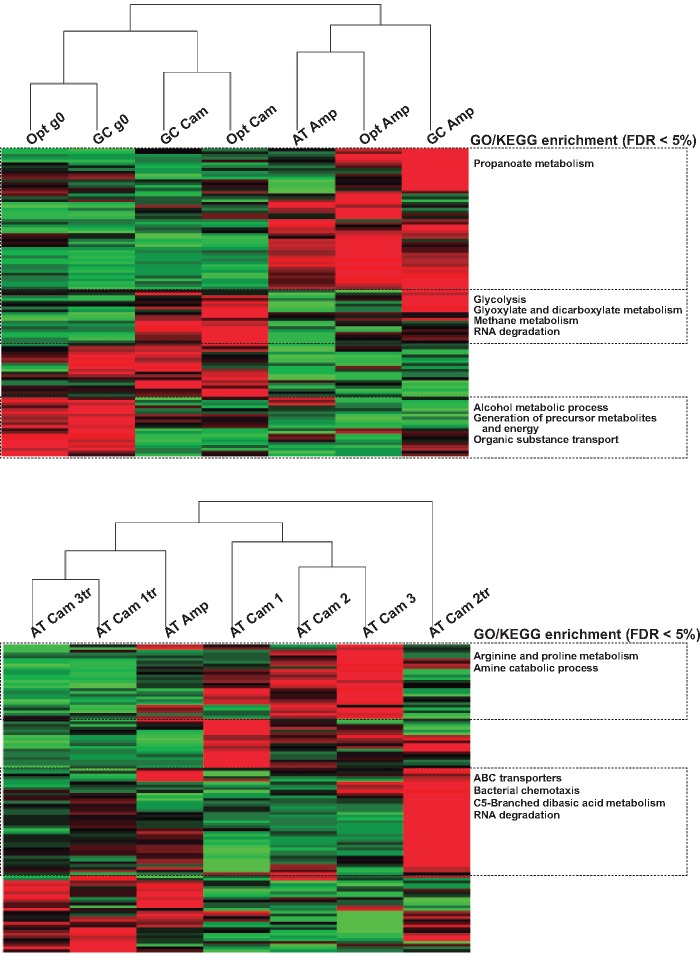
—Comparative proteomics and unsupervised clustering of the proteome profiles in bacterial populations. For both analyses, the AT-*cat* generation zero bacteria served as reference for protein levels. Each horizontal line within a red-to-green gradient reflects the lower-to-higher levels of an individual protein. In the right boxes, enriched functional categories in either the KEGG and in the GO repositories are given for horizontal clusters. (*A*) Comparison of ancestral populations and evolved populations. Populations have been pooled within *gene version* × *antibiotic* of selection combination (bacterial lines carrying the *AT-cat* gene were not included because of methodological constrains). (*B*) For bacterial lines carrying the *AT-cat* gene, comparison of evolved population under ampicillin (Amp) or chloramphenicol (Cam) with ancestral bacteria transformed with plasmids extracted from the evolved populations (labeled as “*tr*”).

Extensive global changes of the cellular proteomic profile were observed, the main driver being antibiotic selection. Additionally, changes were associated with both the plasmid and the chromosome. Unsupervised cluster analysis of the results of a first differential quantitative proteomics experiment (comparing ancestral to evolved populations at g1000) revealed that populations clustered by selection history, yielding three groups (fig. 4*A*): generation zero (i.e., reference starting point before selection), populations selected in chloramphenicol and populations selected in ampicillin, with differences between populations carrying the different versions of the *cat* gene being of smaller amplitude. Interestingly, the three ancestral populations transformed with the three *cat* versions did not display the same proteomic profile, meaning that introduction of synonymous genes impacts differentially the cellular proteome. Such impact of the CUP of a few genes on an important part of the proteome has recently been shown in another study in *E. coli* (Frumkin et al. 2018). Cluster analysis for the second differential quantitative proteomics experiment showed that evolved populations presented a different proteomic profile from the ancestral bacteria transformed with the corresponding evolved plasmid: although the three populations carrying the *cat*-AT and evolved in chloramphenicol clustered together (labeled as ATCam1-3 in fig. 4*B*), ancestral bacteria transformed with plasmids extracted from the evolved populations (labeled as ATCam1-3tr in fig. 4*B*) did not cluster together nor were closer to the respective evolved population from which the plasmids originated (e.g., ATCam1 did not cluster with ATCam1tr in fig. 4*B*). This means that evolutionary changes in the proteome were far from being fully determined by the evolved plasmid.

Functional analysis of the distribution of proteins with altered levels revealed that the largest differences were in the nucleoside and in the carbohydrate metabolism pathways. Some changes seem to be global responses to experimental evolution as they occurred in both populations evolved in ampicillin and in chloramphenicol, such as the increase in levels of enzymes involved in pyrimidine metabolism (*P* = 7.50 × 10^−10^ and 7.37 × 10^−8^ for selection in ampicillin and chloramphenicol, respectively), or in alanine, aspartate and glutamate metabolism (*P* = 5.00 × 10^−3^ and 4.79 × 10^−2^ for selection in ampicillin and chloramphenicol, respectively). Other changes were likely specific responses to the different antibiotics, such as the decrease in ABC transporters in populations selected in chloramphenicol (*P* = 7.19 × 10^−7^) or the increase in enzymes of the purine metabolism (*P* = 6.39 × 10^−3^) and of the pantothenate and CoA biosynthesis (*P* = 4.63 × 10^−2^) in populations selected in ampicillin.

**Fig. 5.**
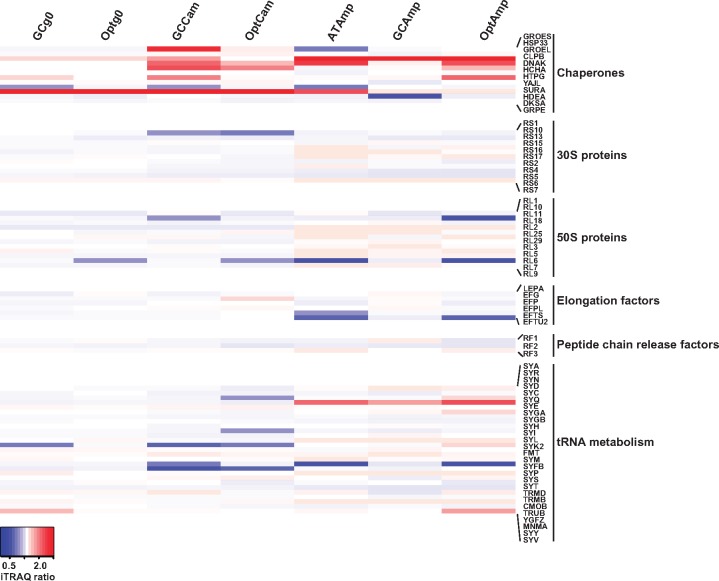
—Heatmap of the relative quantities of candidate proteins in ancestral and evolved populations. Fold changes are expressed relative to the levels in the AT-*cat* g0 population, in the gradient blue-white-red. Intense colors indicate fold changes significantly different from 1.

We analyzed further changes in individual protein levels. For proteins encoded in the plasmid, increased levels of CAT protein in populations selected under chloramphenicol and of BLA in populations selected under ampicillin have been extensively described elsewhere (Bedhomme et al. 2017), and will not be discussed here. Additionally, for proteins encoded in the bacterial chromosome we observed a relevant increase in the levels of proteins involved in the chaperone system, ribosomal function, translation and aminoacyl-tRNA synthesis (fig. 5). For the chaperone systems changes in protein levels seemed to be differentially driven by the antibiotic stress as well as by the expression of the different versions of *cat*: all lines evolved under ampicillin showed an increase in GroEL levels (median 4.06 [2.27–7.29, 95% CI]), whereas for CAM-selected lines this increase was only significant for GC-*cat* lines (1.35 [1.15–1.59, 95% CI]); for ClpB, levels were significantly increased for OPT-*cat* AMP (1.63 [1.28–2.08, 95% CI]) as well as for GC-*cat* CAM and OPT-*cat* CAM (1.61 [1.31–1.99, 95% CI] and 1.31 [1.16–1.48, 95% CI], respectively); levels for DnaK followed the same pattern as those of ClpB, and were significantly increased for OPT-*cat* AMP (1.29 [1.16–1.43, 95% CI]) as well as for GC-*cat* CAM and OPT-*cat* CAM (median 1.64 [1.51–1.79, 95% CI] and 1.45 [1.30–1.73, 95% CI], respectively). Of note, for all three AT-*cat* populations evolved in chloramphenicol, chaperone levels were systematically higher in the populations transformed with the evolved plasmids than in the respective evolved populations: 1.80 (1.38–2.35, 95% CI) versus 1.15 (0.97–1.38, 95% CI) for GroEL; 1.74 (1.56–1.95, 95% CI) versus 0.96 (0.88–1.04, 95% CI) for DnaK; and 3.01 (1.51–5.99, 95% CI) versus 1.71 (0.99–2.94, 95% CI) for HdeA. This increase matches well the increased CAT protein levels in the populations transformed with the evolved plasmids compared with the evolved populations from which their plasmids derive (supplementary table S4, Supplementary Material online). Higher expression levels of the different chaperones may be a response to increased protein misfolding—for example, by the GroEL system—and/or because a need of solubilization and cleaning of protein aggregates—for example, by the ClpB system.

The levels of most 30S and 50S ribosomal proteins were largely unaffected by the antibiotic selection regimes or by the expression of the different *cat* versions. This is possibly not surprising given the strict and concerted equimolarity needed to assemble the complex ribosomal machinery. The sole consistent exception was the significant decrease in the levels of the 30S ribosomal protein S1, product of the *rpsA* gene, for all populations evolved in chloramphenicol (median 0.73 [0.66–0.82, 95% CI; *P* = 1.01 × 10^−5^]). The 30S S1 protein is a part of the trans-translation machinery, engaged in protein tagging and ribosome rescue in stalled translation (Dulebohn et al. 2007), a frequent situation for mRNAs using rare codons.

Differential quantitative proteomics results also revealed large changes in a number of individual proteins involved in stress response. Thus, ampicillin selection lead to changes in the universal stress protein systems: for class I of universal stress protein all populations evolved in ampicillin showed a significant increase in UpsE levels (median 1.35 [1.16–1.58, 95% CI; *P* = 0.0014]), accompanied by a consistent decrease in UpsA levels (median 0.86 [0.57–1.30, 95% CI; *P* = 0.26]); whereas universal stress proteins class II displayed decreased levels for UpsG and UpsF (respective median values 0.49 [0.35–0.70, 95% CI]; *P* = 0.0049; and 0.45 [0.20–1.02, 95% CI]; *P* = 0.052). For the YgiW protein, also involved in stress response, we observed a systematic increase of protein levels in all populations selected in chloramphenicol (median value 2.50 [1.69–3.68, 95% CI; *P* = 9.5 × 10^−^^3^]), mirrored by a substantial albeit nonsignificant decrease in populations selected in ampicillin (median value 0.58 [0.03–8.8, 95% CI; *P* = 0.48]).

Finally, additional large changes in particular protein levels were also observed, although they were not necessarily accompanied by changes in the rest of the proteins of their metabolic/signaling context and their interpretation remains cryptic. This is the case of the significant increase in PykF pyruvate kinase I levels exclusively for all populations selected in ampicillin (median value 1.81 [1.42–2.31, 95% CI; *P* = 3.7 × 10^−^^4^]), whereas no changes were observed in the PykA pyruvate kinase II levels (median value 0.79 [0.61–1.02, 95% CI; *P* = 0.069]).

## Discussion

We have analyzed in this work the genotypic and proteomic changes occurring on an evolutionary short-time scale after the horizontal acquisition of synonymous versions of an antibiotic resistance gene. In many instances the genomic and proteomic results can be interpreted in terms of modifications that may have an adaptive value. In other cases, however, the biological meaning of the experimental evolution outcome remains elusive. We have interpreted the results in terms of the potential adaptive value of the different mutations and genotypic changes that we identify, first to the overall experimental conditions, then to the presence of antibiotics, and finally to the different versions of the *cat* gene. We have then focused on the parallel emergence of large genomic changes and of mutator phenotypes in several lines during the experimental evolution protocol. Finally, we have aimed at integrating all results under the perspective of understanding the influence of HGT on the geno-phenotypic landscapes available to evolution.

### Mutational Patterns Associated with Adaptation to Experimental Conditions

#### Changes in High-Level Regulators

Parallel mutations in global regulators are a common finding in evolve and resequence approaches (Conrad et al. 2014; Long et al. 2015). Essential and key regulator genes were also involved in adaptation to the culture conditions in our experimental evolution setup. This is the case of *arcA*, a global regulator of *E. coli*, mutated in ten out of the 18 populations (table 1). Global regulators are defined as regulators influencing many other regulators and genes by transcription-specific interactions, and capable of sensing a large number of environmental changes (Martı´nez-Antonio and Collado-Vides 2003). ARCA regulates genes involved in redox metabolism (Iuchi and Lin 1988), and mutations in *arcA* have been repeatedly reported in experimental evolution populations adapting to glucose-limited media (Plucain et al. 2014) and to rich media such as lysogeny broth (LB) (Plucain et al. 2014; Saxer et al. 2014). Ten of the eleven *arcA* mutations identified in the present study are located in the receiver domain of the ARCA protein, whereas *arcA* mutations identified by Plucain et al. (2014) are spread over the two protein domains. Because of the multiple effects that mutations in global regulators may bring along, it has been suggested that mutations in global regulators might represent a one-step adaptation path to novel complex environments (Saxer et al. 2014), and we interpret that the mutations we identified in *arcA* may contribute to the adaptation of our populations to our experimental protocol involving growth in LB.

#### Disruption in Intermediate Metabolism Genes

During experimental evolution, certain nonessential genes have been mutated and often rendered nonfunctional or truncated by deleterious mutations, independently of the selection conditions and of the *cat* version. We interpret that such changes may underlie global adaptation to the particular experimental environment, most likely the specific growth conditions. A characteristic example (see table 1) is *fruA* (EC 2.7.1.202), involved in fructose transport (Prior and Kornberg 1988). In line with this gene-specific disabling mutational load, the levels of the FRUA protein were largely decreased in the evolved cell lines, for example, decreased by 70% in AT-Cam lines (where the three of them had accumulated mutations) or by 50% in the AT-Amp lines (where two of the three lines had accumulated mutations). It is important to note that the LB medium used to grow *E. coli* does not contain fructose. The *fruA* expression shutdown is thus clearly compatible with the absence of phenotype for the decrease levels of FRUA for *E. coli* growing on LB (Gerdes et al. 2003; Baba et al. 2006). This nonessentiality is further highlighted by the fact that the active fructose transporter is a multimer containing units of FRUA and FRUB, and the *fruB* gene is disrupted in our *E. coli* genetic background (Durfee et al. 2008). This means that the expression of *fruA* alone would not suffice to restore fructose import function, should this sugar have been present in the medium. Finally, this biological context helps understand the largely deleterious nature of the mutations accumulated in *fruA*: among the nine mutations detected five ablate protein expression by disrupting the open reading frame, three are largely nonconservative amino acid changes, and only one is an A > V conservative one (table 1).

### Mutational and Proteomic Patterns Associated with Antibiotic Treatments

Chloramphenicol acts by interfering with the protein synthesis machinery whereas ampicillin hampers cell-wall synthesis. The sustained presence of either antibiotic imposes a strong and differential selective pressure that drives selection of different sets of genes.

#### Compensation of Chloramphenicol Off-Target Effects

The most cogent example for a gene under strong selective conditions in the presence of chloramphenicol is *adhE* (acetaldehyde/alcohol dehydrogenase EC 1.1.1.1/1.2.1.10) (Goodlove et al. 1989). In *E. coli*, ADHE allows bacterial growth through alcohol fermentation (Trinh et al. 2011). Chloramphenicol decreases ADHE catalytic activity by binding the enzyme outside the active site (Ghenbot and Weiner 1992; Allali-Hassani and Weiner 2001). In our experimental evolution setup, the *adhE* gene was mutated in all populations selected in presence of chloramphenicol. We observed a high level of parallel evolution, with two mutations (A392V and F583L) appearing in three different populations, and with one site being mutated in two different populations (A626V, A626T). Only two of the mutations evolved showed matches to sequences in GenBank: three *Yersinia*, *Shigella*, and *Salmonella* isolates (A626V), and one *Salmonella* isolate (D672N). We interpret that the observed mutations are adaptive and that they may restore the enzyme activity in the presence of chloramphenicol, allowing for ethanol fermentation, because 1) none of the mutations was disruptive, 2) all nonsynonymous mutations were between chemically compatible amino acids, and 3) we did not observe differences in protein levels between bacterial lines under different selection pressures.

#### Potential Adaptation to the Presence of Ampicillin through Changes in Intermediate Metabolism

We observed mutations accumulating in the *sucC* gene in eight lines, six of them evolving in ampicillin (table 1). The *E. coli* succinyl-CoA synthetase (EC 6.2.1.5) is a heterotetramer, with two subunits encoded by the *sucD* gene and two subunits encoded by the *sucC* gene. In the Krebs cycle, this enzyme catalyzes the reversible reaction (succinyl-CoA + NDP ⇆ succinate + CoA + NTP) generating ATP or GTP. We identified a total of ten different mutations in *sucC*, nine of them nonsynonymous. The E350G mutation displayed high parallelism and appeared independently in two AT_Cam and one AT_Amp lines. All nine mutations corresponded to strongly conserved amino acids. Indeed, there is no single entry within Gammaproteobacteria in GenBank displaying any of these changes. All these mutations mapped to the interface between alpha and beta subunits in the SUCC–SUCD heterotetramer (Fraser et al. 1999). Two of the mutations (Q247R and W248R) occurred in sites known to modify the dimer–dimer interaction. The mutational profiles suggest that the gross catalytic activities of the SUCC–SUCD heterotetramer are not essentially modified, but that the fine-tuning allosteric control through substrate synergism, characteristic of this enzyme (Birney et al. 1997), might be modified. Nevertheless, the effects of the mutations in the *sucC* gene on the quaternary structure and function of the enzyme remain cryptic, although the high level of target parallelism and the absence of similar mutations in the databases strongly suggest that they do confer an adaptive value under both antibiotics.

At the functional level, we observe for the SUCC–SUCD heterotetramer a concerted variation in the levels for SUCC and SUCD (*R*^2^ = 0.921), so that changes in monomers levels likely reflect changes in the functional SUCC–SUCD heterotetramer. Overall SUCC–SUCD levels were increased (20–50%, respectively) in populations selected in ampicillin, whereas they were decreased in populations selected in chloramphenicol (around 50% for both proteins). We interpret that these changes reflect the differential demands for succinyl-CoA synthetase activity under either antibiotic. Very interestingly, variations in succinyl-CoA synthetase matched well variations in the levels of isocitrate dehydrogenase (*aceA*; EC 4.1.3.1; isocitrate ⇆ succinate + glyoxylate), an enzyme that branches-off the Krebs cycle and engages into the glyoxylate cycle. Levels of this protein were increased between 40% and 60% in populations selected under ampicillin and decreased 40–20% in populations selected under chloramphenicol. The glyoxylate cycle shortcuts the Krebs cycle by driving carbon flux from fatty acids into gluconeogenesis. A similar upregulation of genes involved in the glyoxylate cycle has been described in *Acinetobacter oleivorans* when exposed to ampicillin but not when exposed to antibiotics targeting protein synthesis (Heo et al. 2014). Our results suggest that there exists a differential adaptation controlling the metabolic flows through the Krebs cycle and through the glyoxylate cycle when bacteria are grown in the presence of ampicillin or of chloramphenicol.

#### Potential Adaptation to the Presence of Ampicillin through Changes in Essential Ribosomal Genes

A puzzling finding is the parallel selection for early stop mutations in the *rpsG* gene, encoding for the S7 protein in the 30S ribosome. The parallel mutations occurred in two OPT-*cat* and two GC-*cat* populations, all selected in ampicillin (see table 1). In these four cases, the *rpsG* gene underwent point mutations in exactly the same nucleotide leading to a 22 amino acid truncation. This polymorphism in the S7 protein length is actually known, the long version being characteristic of *E. coli K* and the short version of *E. coli B*. The mutations obtained produce a S7B-like protein (Reinbolt et al. 1979). Functional differences between the two protein forms are not documented. In quantitative proteomics we observed a trend toward higher levels of S7 protein in lines selected in the presence of ampicillin, independently of the version of the *cat* gene (median fold change 3.92), but the peptide coverage was not high enough to assess significant differences.

#### Proteomic Changes Associated with Antibiotic Treatment

In the evolved populations, changes in the proteome are largely driven by the selection treatment (fig. 4*A*). The increase in the GroEL or CplB chaperones, the decrease in S1 protein (fig. 5), and the decrease in pyrimidine, alanine, aspartate and glutamate metabolism, seem to be a general response to stress selection, independently of the biochemical nature of the antibiotic activity. Just as the specific mutations described above, some consistent proteomics trends appear to be different for selection under chloramphenicol or under ampicillin. This is the case of the increase in purine metabolism in cells selected in ampicillin. Selection in ampicillin triggered also a significant decrease in the universal stress protein G, which is usually increased in response to a number of stressors (Bochkareva et al. 2002). Evolution under chloramphenicol selected for a significant increase of the YigW stress protein, which is involved in hydrogen peroxide and cadmium stress responses (Lee et al. 2009). Despite specific changes for populations evolved in chloramphenicol, no clue of evolution of alternative resistance mechanisms could be detected. Indeed, none of the efflux pumps presented a significant increase and on the contrary, ABC transporters presented a significant decrease for populations selected in chloramphenicol.

### Mutational Patterns Associated with One of the *cat* Versions

Several genes were the targets for more mutations in the lines containing the AT-*cat* gene than in the lines containing other *cat* versions. The best example is *spoT*, mutated in four populations carrying AT-*cat* and one carrying the Opt-*cat* (table 1). *spoT* is an essential gene (Baba et al. 2006) with a central role in the biosynthesis of ppGpp: the SPOT protein has both ppGpp hydrolase (EC 3.1.7.2) and ppGpp synthase (EC 2.7.6.5) activities, which respectively degrade and synthesize ppGpp (Hernandez and Bremer 1991; Somerville and Ahmed 1979). Fine regulation of ppGpp levels is crucial to the cell, as this small molecule serves as an alarmone setting off the stringent response under different starvation conditions (Hauryliuk et al. 2015). The stringent response shuts down ribosomal synthesis and drives cellular energy into intermediate metabolism. SPOT may additionally have an effect on translation as it binds directly to OBG, a GTPase involved in ribosome assembly and that is also an effector of the stress response (Wout et al. 2004). All six mutated residues correspond to strictly conserved amino acid positions in SPOT, and none of the retrieved mutations matched any *Enterobacteriaceae* entry in GenBank. The AT-*cat* gene poses the strongest translational stress in our setup, induced by the need of sustained expression of this gene with an important CUP mismatch. We propose that the differential increase in fixed *spoT* mutations in the AT-*cat* lines evolved under the presence of chloramphenicol may indeed be an adaptive response to such strong ribosomal stress. CUP mismatch and amino acid starvation have similar cellular consequences, for example ribosome stalling, and mutations in *spoT* could reduce the transduction of the stress signal into a downregulation of translation, growth, and division.

Although we have tried to discern the potential adaptive value for the mutations differentially enriched in cell lines carrying different versions of the cat gene, we are still far from understanding all the differential mutational patterns that we identify. For the AT-*cat* populations, this is the case for the excess of nonsynonymous mutations in the *putP* gene (encoding for a proline importer) or of the probably disruptive mutations in the *slt* gene (encoding for a murein transglycosylase). For the OPT-*cat* populations, this is the case for the excess of nonsynonymous mutations in the *fadE* gene (encoding for an acyl-CoA dehydrogenase, limiting step of beta-oxidation).

### Parallel Evolution between Evolved Populations

#### Parallel Emergence of Mutator Phenotypes

A striking outcome of the genomic analysis of the evolved populations is the high proportion of populations that became mutators. The mutator phenotype was due in all cases to an IS10 insertion in *mutS* or *mutL*, essential genes of the MMRS surveillance system. Mutations in such genes are one of the main sources of mutator phenotypes (Sniegowski et al. 1997). It is actually quite usual to obtain mutator populations when performing experimental evolution (Sniegowski et al. 1997; Long et al. 2015; Tenaillon et al. 2016), and screening of clinical and natural isolates established that around 2% of them present a mutator genotype (LeClerc et al. 1996; Oliver 2000; Denamur et al. 2002). Most mutations are deleterious, such that mutators are likely to have an average negative effect. However, the proportion of mutations with a positive effect on fitness increases when a population faces a new environment (Martin and Lenormand 2006), and in clonal populations a mutator genotype can hitch-hike with the beneficial mutations it triggers (Notley-McRobb and Ferenci 2000; Ferenci 2003). For these reasons, mutators have been predicted to have a transient advantage by increasing the adaptation rate (Tenaillon et al. 1999) as demonstrated in some specific cases (Notley-McRobb and Ferenci 2000). By dating the appearance of the mutator genotype responsible for mutation increase in each population, we were able to show that mutators appeared earlier for populations evolved in presence of chloramphenicol than for populations evolved in presence of ampicillin (*χ*^2^_1_=5.07, *P* = 0.024; see supplementary material and supplementary fig. S2, Supplementary Material online). Because the initial stress imposed by chloramphenicol is stronger than the one imposed by ampicillin, our results are consistent with the hypothesis that mutators are advantageous in stressful environments. The high mutator frequency in our populations is partly explained by the presence in the ancestral genome of an IS10 insertion sequence, known to have a very high transposition rate—around 10^−4^ per cell per bacterial generation—(Shen et al. 1987), which accounts also for the high number of novel IS10 insertions detected in the evolved genomes (see supplementary table S1, Supplementary Material online). Our populations were thus probably very prone to become mutators by IS10 insertion in MMRS genes. Although insertion sequences are the smallest mobile elements and are often perceived as parasitic sequences with deleterious effects, previous experimental evidence (Stoebel and Dorman 2010; Gaffé et al. 2011) together with the results shown here suggest that their insertions can trigger fitness increases, by disrupting stress response regulators or, as in our case, by inducing mutator phenotypes, and thus changing the course of adaptive evolution.

#### Parallel Emergence of Large Genomic Duplications

Another characteristic pattern of our experimentally evolved lineages is the relatively frequent occurrence of large duplications, presenting for some of them a high level of parallelism (fig. 2). Large duplications are known to occur at high frequency not only in bacterial genomes (Andersson and Hughes 2009) but also in eukaryotes (Yona et al. 2012; Szamecz et al. 2014; Kalapis et al. 2015). Duplication rates vary along the genome, for example, between 6 × 10^−5^ and 3 × 10^−2^ per genome per generation in *S.typhimurium* (Anderson and Roth 1981). Duplications mainly occur between homologous sequences, such that the duplication rate of a specific sequence depends on its relative position in terms of chromosomal distance (Andersson and Hughes 2009). The high level of parallelism in our data could thus partly reflect that certain duplications occur at a high rate. In one case we were actually able to determine that the duplication occurred between ribosomal RNA operons, namely *rrnA* and *rrnC* (duplication between 4,038,000 and 4,139,000 approximately). The seven *rrn* operons of *S. typhimurium* (genome structure close to *E. coli*) were the first highly homologous regions identified as recombination points generating duplications (Anderson and Roth 1981). More recently, it was established that high rates of duplication lead, in the absence of selection, to a steady-state frequency of cells carrying a specific duplication in a population (Reams et al. 2010; Adler et al. 2014). It is thus very likely that the populations from which we started the experimental evolution already carried duplications at very low frequencies. Such diversity represents a form of standing variation upon which selection can immediately act. Large duplications have recurrently been detected in populations adapting to novel environments, in a broad sense (reviewed in Andersson and Hughes 2009). Selective advantage of duplications is thought to come from an effect on gene dosage and on the associated changes in gene expression level of key genes. The specific genes whose amplification provides an advantage are easy to identify in some cases, as for example when they are antibiotic resistance genes (Laehnemann et al. 2014; Hjort et al. 2016). In other cases, identifying them requires enrichment analyses to detect functions that are overrepresented in the duplicated genes (Kalapis et al. 2015). Here, a hypothesis to explain the advantage of the duplications observed was that they might modify the tRNA gene pool thereby increasing the match to the CUP in the introduced *cat* gene versions. We have explored both adaptive hypotheses for the emergence/conservation of long duplications in our experimentally evolved populations and none of them could be validated by our data: we did not observe any consistent pattern of functional enrichment nor evidence for directional enrichment of tRNA genes.

Fixation of large genomic duplications is likely a swift but rough adaptation path. Swift, because duplications occur at a much higher rate than the individual point mutations that would produce a similar increase in gene product activity (Reams et al. 2010); rough, because the increase in fitness provided by the upregulation of certain genes is mitigated by the costs of overexpression of other duplicated genes that may not provide any advantage. It can thus be envisioned that the selective advantage of duplications may be transient, and that, on the long run, refinements will appear and may eventually lead to the duplication loss. Such an adaptation path spanning *duplication-refinement-duplication loss* cycles has been identified in yeast populations adapting to heat (Yona et al. 2012). Consistent with this view, we identify populations with specific large duplications present at generation 458 but not anymore at generation 1,000 (fig. 2), whereas such a pattern was very rarely observed for point mutations.

## Conclusion

Our results, together with other recent studies (Lind et al. 2010; Bershtein et al. 2015), indicate that HGT contributes to bacterial genome evolution beyond the provision of new pieces and new functions: the horizontal transfer of a gene, particularly if its CUP differ from those in the receiving genome, induces selection pressures leading to a diversity of genomic changes and a widely modified proteomic profile. At first sight these changes can seem poor evolutionary solutions because they come with obvious high costs: accumulation of deleterious mutations in the case of mutators, pleiotropic effects in the case of mutations in global regulators and disadvantageous changes in gene dosage in the case of duplications. Notwithstanding, a likely explanation for their success is their high probability to be the first to occur because of their high mutational potential (Long et al. 2015). After subsequent evolution, bacteria carrying the transferred gene may reach a similar fitness as their ancestors but through different pathways. Our experimental protocol applied extreme codon maladaptation, and the gene introduced was essential for the survival in presence of chloramphenicol. Consequently, the selection pressure was strong, and likely stronger than in most natural HGT cases. However, our experimental setting is not unrealistic, as HGT is the main spreading mode for certain antibiotic resistance genes and can happen between organisms with very distinct CUP. HGT may thus be a powerful mechanism pushing bacteria to explore new ways of functioning. Ultimate fitness improvements can thus arise not only from the acquisition of a ready-to-use function but also from innovation leaps resulting from the novel selection pressures that the presence of the transferred gene exerts.

## Supplementary Material

Supplementary data are available at *Genome Biology and Evolution* online.

## Supplementary Material

Supplementary Data
