# Supplementary material for: Evolutionary Changes after Translational Challenges Imposed by Horizontal Gene Transfer
Source: Genome Biol Evol. 2019 Feb 11;11(3):814–31. doi: 10.1093/gbe/evz031 (PMC6427688; doi:10.1093/gbe/evz031)
Supplement: Supplementary Data [file evz031_supp.zip › Supplemental material_GBE_180539.docx]

**Supplemental material and methods**

**i-TRAQ experiment and analyses**

Following the instructions of the supplier (ABSciex), 110 μg of extracted protein per sample were reduced with phosphine and cysteins and blocked with methyl methanethiosulfo-nate followed by addition of trypsin (Promega) at a trypsin:protein ratio of 1:10 (w/w). The digest mixtures were incubated overnight at 37 °C. After trypsin digestion, the peptide mixtures were dried in a speed vacuum. Each sample was re-dissolve in 80 µL of TEAB + ethanol solution (3/7; v/v), with sonication for 10 min, and added to the appropriate iTRAQ reagent vial and vortex thoroughly. Immediately each sample vial was rinsed with additional 20 µL of TEAB + ethanol solution and transfer to the correct iTRAQ reagent vial, and incubated at ambient temperature for 3 hours. After labeling, using the eight mass reporters available, the eight samples were mixed, divided in two equal aliquots and dried. One aliquot was dissolved in 8M Urea/2 M thiourea in water (250 µL) with carrier ampholyte (2% (v/v) 3-10 pH gradient (GE)). Immobiline IPG strips (13 cm; pH gradient 3-10 NL; GE) were placed over the samples and allowed to rehydrate overnight at ambient temperature. The loaded strips were focused at 20°C on an Ettan IPGPhor-II IEF system (GE Healthcare) using 5000 V for 30000 Vh. The strips were then cut into 11 pieces (10 mm) and placed in separate eppendorf tubes. Peptides were eluted from the strips with 75 μL of 0.1% trifluoroacetic acid (TFA) for 15 min, followed by 75 μL of 50% acetonitrile (ACN)/0.1%TFA for 15 min, then with 75 μL of ACN/0.1% TFA for 15 min. The combined eluates for each IPG strip fraction were evaporated via centrifugal vacuum evaporation on a Speedvac and then redissolved in 2% acetonitrile in 0.1% TFA. The extracts were subjected to further cleanup using POROS R2 (Applied biosystems) solid phase extraction before LC-MS/MS, to remove residual overlay oil from the IEF process and other interferents. Then there were applied to a homemade POROS R2 micro columns (ca 10 µL) previously prewashed with 100 µL ACN and equilibrated with 200 µL 2%ACN/0.1% TFA. The bounded peptides were washed with 100 µL 2%ACN/0.1% TFA and eluted with 250 µL each of 30% acetonitrile/0.1% TFA, 70% acetonitrile/0.1% TFA and 100% acetonitrile/0.1% TFA and the combined eluates were evaporated in vacuo and redissolved in 25 μL of 2%ACN/0.1% TFA for LC-MS/MS analysis. 5 μl of the sample was loaded onto a trap column (PepMap C18, 300 μm X 5 mm; LC Packings) and desalted with 0.1% TFA at 30 μl/min during 5 min. The peptides were then loaded onto an analytical column (PepMap C18 3 μ 100A, 75 μm X 15 cm, LC Packings) equilibrated in 0.1% formic acid (FA). Elution was carried out with a linear gradient 2–50% of solvent B (95% ACN/0.1% FA) in 90 min at a flow rate of 300 nl/min. The eluted peptides were analyzed with a nano-ESI-Q-TOF mass spectrometer (QSTAR-XL; Applied Biosystems) in an information-dependent acquisition mode, in which a 1-s TOF MS scan from 400 to 2000 m/z was performed, followed by 3-s product ion scans from 65 to 2000 m/z on the three most intense doubly or triply charged ions.

The MS/MS data of all fractions combined were analyzed using ProteinPilot version 2.0 (Applied Biosystems). For ProteinPilot searches, default parameters were used to generate the peak list directly from QSTAR wiff files. The Paragon algorithm of ProteinPilot was used to search the latest Expasy protein database with the following parameters: trypsin specificity and cys-alkylation; taxonomy fixed to *E. coli* (8868 proteins searched); and quantitation iTRAQ 8-plex at peptide level. To avoid using the same spectral evidence in more than one protein, the proteins identified were grouped based on MS/MS spectra, using the Protein-Pilot Progroup algorithm. Thus, proteins sharing MS/MS spectra were grouped, regardless of the peptide sequence assigned. The protein within each group able to explain more spectral data with confidence is shown as the primary protein of the group. Only proteins with a ProteinPilot unused scored above 1.3, which is equivalent to a protein confidence threshold greater than 95%, and for which there were at least one unique peptide match with a confidence>95% were selected and shown in the results. For relative quantification, the bias calculated by Paragon were applied and only those changes having a statistically significant change at P < 0.05 were considered.

For functional annotation, the Gene Ontology (GO) Biological Processes term and the Kyoto Encyclopedia of Genes and Genomes (KEGG) pathway annotations were downloaded from the corresponding repositories (Open Biological Ontologies release 06/2012 MySQL version; KEGG 12/2012). GO terms were assigned to gene symbols after record linkage in which regular expression searches were required. Genes annotated at level 5 or lower in the hierarchy were assigned to level 4, but those also occurring at level 3 were excluded. A similar procedure was applied for annotations at level 4 or lower. Only terms with a frequency above 5% in each cluster set were evaluated.

**Detection of IS10 insertion in *mut* genes for the temporal analysis of mutator appearance.**

*PCR test:* Populations where grown overnight in LB containing the antibiotic under which they had evolved. DNA was extracted using a Purelink microbiome DNA purification kit following manufacturer’s instructions. PCR was performed with primers amplifying around the IS10 insertion point as indicated by the genome sequencing. The primer pairs used for each population are given in the table S5. The PCR program was the following one: 5 minutes at 98º, 30 cycles of 1 minutes at 98º, 30 seconds at 60º and 2 minutes at 72º and final 5 minutes at 72º.

*Statistical analysis:* The temporal dynamics of mutator appearance was analysed by a parametric survival analysis applied to “time to first mutator detection”, with censored data. Five different distributions were fitted to the data and the one with the best AIC was selected. Two separate parametric survival analyses were conducted, one testing for the effect of antibiotic (distribution with the best fit: Frechet) and the other testing for the effect of gene versions (distribution with the best fit: exponential). Antibiotic showed a significant effect (χ^2^_1_=5.07, p=0.024, see figure S1 below) whereas gene version did not have any significant effect.

**Supplementary tables**

**Table S1. Complete list of genomic changes identified by whole genome sequencing of the evolved populations.** Mutations are colour coded with the same code as in Fig 3.

Provided as a separate Excel file.

**Table S2. List of genes mutated in two to four populations sequenced at generation 1000.** The shades of blue in the third column reflect whether 0, 1 or 2 of the replicate populations in each gene version * antibiotic combination were mutated.

| Mutated gene(s) | Antibiotic | *cat* gene version | | |
| --- | --- | --- | --- | --- |
|  |  | AT | GC | OPT |
| *ybeZ* | AMP |  |  |  |
|  | CAM |  |  |  |
| *cpxR recG yjjK* | AMP |  |  |  |
|  | CAM |  |  |  |
| *ebgA ptsA* | AMP |  |  |  |
|  | CAM |  |  |  |
| *caiT dgt glf mraW yhiF yneK yrhC* | AMP |  |  |  |
|  | CAM |  |  |  |
| *nrfG* | AMP |  |  |  |
|  | CAM |  |  |  |
| *ftsI* | AMP |  |  |  |
|  | CAM |  |  |  |
| *fhuB yhjJ* | AMP |  |  |  |
|  | CAM |  |  |  |
| *imp* | AMP |  |  |  |
|  | CAM |  |  |  |
| *astE* | AMP |  |  |  |
|  | CAM |  |  |  |
| *ydbA* | AMP |  |  |  |
|  | CAM |  |  |  |
| *pqiA sbmA* | AMP |  |  |  |
|  | CAM |  |  |  |
| *topA* | AMP |  |  |  |
|  | CAM |  |  |  |
| *ynfM* | AMP |  |  |  |
|  | CAM |  |  |  |
| *cheR rsmC tpx yfhM yfiN* | AMP |  |  |  |
|  | CAM |  |  |  |
| *xdhB* | AMP |  |  |  |
|  | CAM |  |  |  |
| *obgE* | AMP |  |  |  |
|  | CAM |  |  |  |
| *yegH* | AMP |  |  |  |
|  | CAM |  |  |  |
| *arcB lpxM rsxC* | AMP |  |  |  |
|  | CAM |  |  |  |
| *degQ prfB* | AMP |  |  |  |
|  | CAM |  |  |  |
| *polA yqhD* | AMP |  |  |  |
|  | CAM |  |  |  |
| *metQ pcnB yggR yqiG* | AMP |  |  |  |
|  | CAM |  |  |  |
| *acrA* | AMP |  |  |  |
|  | CAM |  |  |  |
| *xdhA* | AMP |  |  |  |
|  | CAM |  |  |  |
| *exbB ydiQ* | AMP |  |  |  |
|  | CAM |  |  |  |
| *yedV* | AMP |  |  |  |
|  | CAM |  |  |  |
| *trpD ygbI yibA ytfN* | AMP |  |  |  |
|  | CAM |  |  |  |
| *paaK rpoB* | AMP |  |  |  |
|  | CAM |  |  |  |
| *purH yacH ydiN yhcD yqiK* | AMP |  |  |  |
|  | CAM |  |  |  |
| *yddA* | AMP |  |  |  |
|  | CAM |  |  |  |

| Mutated gene(s) | Antibiotic | *cat* gene version | | |
| --- | --- | --- | --- | --- |
|  |  | AT | GC | OPT |
| *treB* | AMP |  |  |  |
|  | CAM |  |  |  |
| *frdD* | AMP |  |  |  |
|  | CAM |  |  |  |
| *elaD idnD* | AMP |  |  |  |
|  | CAM |  |  |  |
| *trkH* | AMP |  |  |  |
|  | CAM |  |  |  |
| *yddB yfhK* | AMP |  |  |  |
|  | CAM |  |  |  |
| *atoB cadA dcuA trpC* | AMP |  |  |  |
|  | CAM |  |  |  |
| *proY* | AMP |  |  |  |
|  | CAM |  |  |  |
| *yhcF* | AMP |  |  |  |
|  | CAM |  |  |  |
| *ybaL* | AMP |  |  |  |
|  | CAM |  |  |  |
| *cspD ytfK* | AMP |  |  |  |
|  | CAM |  |  |  |
| *tnaA* | AMP |  |  |  |
|  | CAM |  |  |  |
| *speG* | AMP |  |  |  |
|  | CAM |  |  |  |
| *tktB* | AMP |  |  |  |
|  | CAM |  |  |  |
| *htrE* | AMP |  |  |  |
|  | CAM |  |  |  |
| *fecA* | AMP |  |  |  |
|  | CAM |  |  |  |
| *cyoA* | AMP |  |  |  |
|  | CAM |  |  |  |
| *yahB ynbC* | AMP |  |  |  |
|  | CAM |  |  |  |
| *rbsD* | AMP |  |  |  |
|  | CAM |  |  |  |
| *emrY* | AMP |  |  |  |
|  | CAM |  |  |  |
| *bcsC bglF yqeB* | AMP |  |  |  |
|  | CAM |  |  |  |
| *norR* | AMP |  |  |  |
|  | CAM |  |  |  |
| *fixC narZ uup* | AMP |  |  |  |
|  | CAM |  |  |  |
| *fruK* | AMP |  |  |  |
|  | CAM |  |  |  |
| *dapB fhuD lacY proS ybbP* | AMP |  |  |  |
|  | CAM |  |  |  |
| *arnT barA fusA pdxK sfmA yfjZ* | AMP |  |  |  |
|  | CAM |  |  |  |
| *yoaE* | AMP |  |  |  |
|  | CAM |  |  |  |
| *melB* | AMP |  |  |  |
|  | CAM |  |  |  |
| *bcsB glk lpxB menD modF tdcA tfaR tyrA ycbB ygfT yqiJ zraR* | AMP |  |  |  |
|  | CAM |  |  |  |
| *ygdH* | AMP |  |  |  |
|  | CAM |  |  |  |

**Table S3. tRNA gene pool of the ancestral and of the evolved populations carrying the GC-*cat* gene.** A rare codon is defined as one having a frequency of use inferior to half the expected frequency assuming equal use within synonymous codons families. Symmetrically, a frequent codon is defined as one having a frequency of use superior to double the expected frequency assuming equal use within synonymous codons families. The “Ancestral genome” column gives the numbers of genes of each codon type. Numbers in parenthesis are proportions of the total tRNA gene number. The “evolved genomes” columns give the change in tRNA gene content for each codon type. Numbers in parenthesis are proportions of the total number of changes in each genome.

|  | Ancestral genome | Evolved genome | | | | | | | | | | | |
| --- | --- | --- | --- | --- | --- | --- | --- | --- | --- | --- | --- | --- | --- |
|  |  | Amp1 g458 | Amp1 g1000 | Amp2 g458 | Amp2 g1000 | Amp3 g458 | Amp3 g1000 | Cam1 g458 | Cam1  g1000 | Cam2 g458 | Cam2 g1000 | Cam3 g458 | Cam3 g1000 |
| rare | 22  (0.26) | 1  (1) | 0 | 1  (1) | 1  (1) | 1 (0.11) | 0 | 2 (0.13) | 2 (0.15) | 4 (0.22) | 1 (0.14) | 1 (0.11) | 2 (0.12) |
| medium | 48  (0.56) | 0 | 0 | 0 | 0 | 5 (0.56) | 0 | 7 (0.44) | 5 (0.38) | 10 (0.56) | 3 (0.43) | 5 (0.56) | 9 (0.53) |
| frequent | 16  (0.19) | 0 | 0 | 0 | 0 | 3 (0.33) | 0 | 7 (0.44) | 6 (0.46) | 4 (0.22) | 3 (0.43) | 3 (0.33) | 6 (0.35) |

**Table S4. CAT protein level changes.** Relative quantity of CAT protein and 95%CI in the evolved ATCam populations and populations of ancestral bacteria transformed with plasmids extracted from the corresponding evolved ATCam populations (noted “transformed populations”). The quantities were determined in the second iTRAQ analyses. All values are expressed relatively to the AT-rich-cat g0 population (p-values for all comparisons with the reference are below 0.002).

|  | *ATCam1* | *ATCam2* | *ATCam3* |
| --- | --- | --- | --- |
| Evolved populations | 3.60  (2.28 – 5.68) | 2.60  (1.80 – 3.77) | 2.23  (1.55 – 3.23) |
| Transformed populations | 5.81  (3.41 – 9.89) | 7.73  (2.61 – 22.94) | 3.17  (1.98 – 5.06) |

**Table S5. Primers used to detect IS10 insertions in *mutL* and *mutS*.**

| IS10 insertion point detected | Primer pair | Populations tested |
| --- | --- | --- |
| mutL (977) | F: TGGAGATCGACCCACATCAG  R: GCCGGTTCTGCAAAGTGATT | OptAmp2, OptCam2, OptCam3 |
| mutL (1423) | F: AATCACTTTGCAGAACCGGC  R: CGCTCATCAGATTTCGTGCA | ATAmp2, ATAmp3 |
| mutS (1552) | F: CCCATCAACTACATGCGTCG  R: GCGGTTTCAGTCATCTCCAC | ATCam2, ATCam3 |
| mutS (1999) | F: TTGATCATCACCGGTCCGAA  R: GTTAGCGACGCCTTCCATTT | OptAmp3, OptCam1 |
| mutS (2463) | F: AAATGGAAGGCGTCGCTAAC  R: ACACCAGGCTCTTCAAGCGA | GCAmp2, GCCam2 |

**Supplemental figures**

**Figure S1. Summary of the characteristics of the three gene versions and the derivation of experimental evolution populations.** CAI_HEG_ is the Codon Adaptation Index (Sharp & Li 1987) calculated using a set of highly expressed genes in *E.coli* as reference (Puigbò et al. 2008).


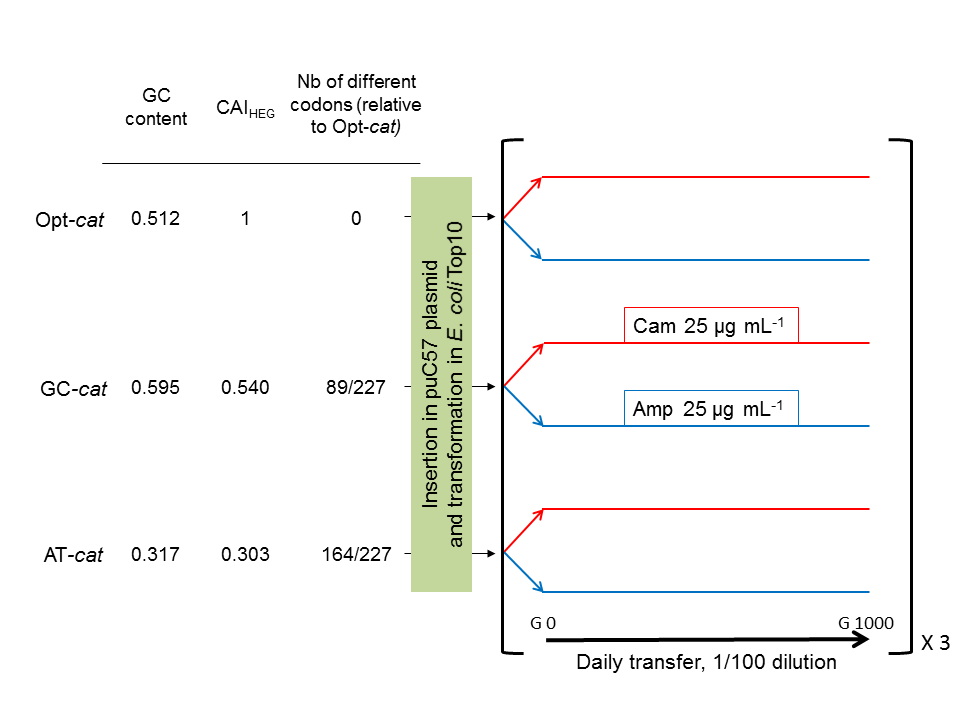


**Figure S2. Temporal dynamic of mutator appearance in experimentally evolved populations.** The presence of an IS10 insertion in *mutL* or *mutS* was detected by PCR. The absence of mutator is represented by a thin black line, a mix of mutator and non-mutator is represented by an orange thick interrupted line and all mutator populations are represented by continuous thick orange lines.

**
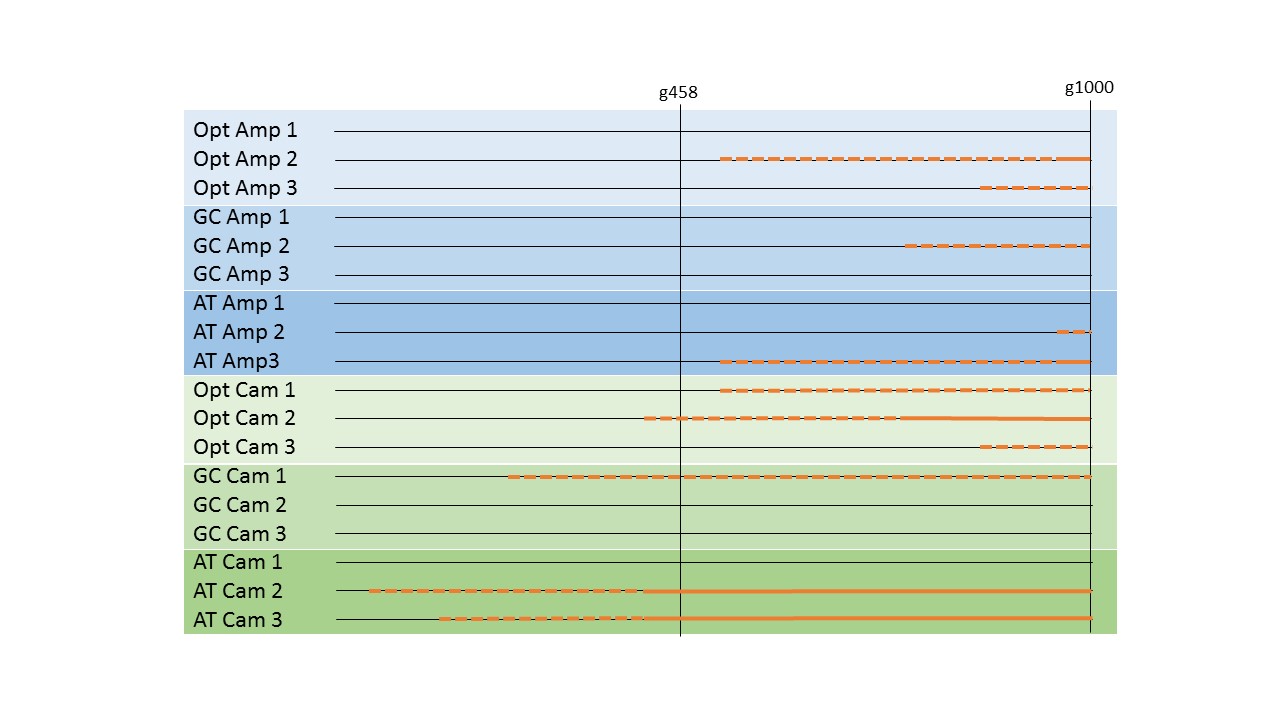
**

**Literature cited:**

Puigbò P, Romeu A, Garcia-Vallvé S. 2008. HEG-DB: a database of predicted highly expressed genes in prokaryotic complete genomes under translational selection. Nucleic Acids Res. 36:D524–527. doi: 10.1093/nar/gkm831.

Sharp PM, Li W-H. 1987. The codon adaptation index-a measure of directional synonymous codon usage bias, and its potential applications. Nucleic Acids Res. 15:1281–1295. doi: 10.1093/nar/15.3.1281.
